# Bacterial vaginosis is associated with uterine cervical human papillomavirus infection: a meta-analysis

**DOI:** 10.1186/1471-2334-11-10

**Published:** 2011-01-11

**Authors:** Evy Gillet, Joris FA Meys, Hans Verstraelen, Carolyne Bosire, Philippe De Sutter, Marleen Temmerman, Davy Vanden Broeck

**Affiliations:** 1 International Centre for Reproductive Health (ICRH), Ghent University, Ghent, Belgium; 2 Department of Gynaecology, Vrije Universiteit Brussel, Brussels, Belgium; 3 Department of Applied mathematics, biometrics and process control, Faculty of Bioscience Engineering, Ghent University, Ghent, Belgium; 4 Department of Obstetrics and Gynaecology, Ghent University Hospital, Ghent, Belgium

## Abstract

**Background:**

Bacterial vaginosis (BV), an alteration of vaginal flora involving a decrease in Lactobacilli and predominance of anaerobic bacteria, is among the most common cause of vaginal complaints for women of childbearing age. It is well known that BV has an influence in acquisition of certain genital infections. However, association between BV and cervical human papillomavirus (HPV) infection has been inconsistent among studies. The objective of this meta-analysis of published studies is to clarify and summarize published literature on the extent to which BV is associated with cervical HPV infection.

**Methods:**

Medline and Web of Science were systematically searched for eligible publications until December 2009. Articles were selected based on inclusion and exclusion criteria. After testing heterogeneity of studies, meta-analysis was performed using random effect model.

**Results:**

Twelve eligible studies were selected to review the association between BV and HPV, including a total of 6,372 women. The pooled prevalence of BV was 32%. The overall estimated odds ratio (OR) showed a positive association between BV and cervical HPV infection (OR, 1.43; 95% confidence interval, 1.11-1.84).

**Conclusion:**

This meta-analysis of available literature resulted in a positive association between BV and uterine cervical HPV infection.

## Background

Bacterial vaginosis (BV) is the most prevalent cause of abnormal vaginal discharge, affecting women of reproductive age [1]. This infestation is characterized by a loss of indigenous (hydrogen peroxide-producing) *Lactobacillus*-predominant vaginal microflora, and a concurrent massive overgrowth of anaerobic bacteria. The most common include *Gardnerella vaginalis*, *Mobiluncus species*, *Prevotella species*, *Mycoplasma hominis *and *Atopobium vaginae *[2]. At least 50% of patients have no symptoms [3]. In the other half, it most often manifests clinically as a thin homogenous vaginal discharge, a vaginal pH of more than 4.5, presence of 'clue cells', and an amine odour after addition of 10% of potassium hydroxide [1,2].

The etiopathogenesis of this condition remains subject of debate. Some risk factors have been associated with BV, including cigarette smoking, use of intrauterine devices, frequent vaginal douches, multiple sexual partners, early age at first intercourse, and black ethnicity [4,5]. BV has been shown to increase the risk of obstetric and gynaecologic complications such as preterm labour and delivery, chorioamnionitis, post-caesarean endometritis, postabortion pelvic inflammatory disease, and cervicitis [6,7]. Moreover, BV has been associated with many sexually transmitted infections (STIs), including infection with *Chlamydia trachomatis*, *Neisseria gonorrhoeae*, HSV-1 and 2, and an increased risk of HIV acquisition [4,8,9]. The leading hypothesis concerning these associations is that absence of protective lactobacilli increases biological susceptibility of acquiring an STI upon exposure. However, the temporal nature of the association between BV and acquisition of STIs remains an ongoing discussion. Although there is a large bulk of evidence favouring the plausibility that BV also incurs an elevated risk for human papillomavirus (HPV) acquisition, this remains a matter of debate.

It is well known that infection with oncogenic HPV, a sexually transmitted DNA virus, is the central etiological agent in the development of cervical cancer. Persistent HPV infection is a prerequisite for progression to high-grade lesions [10]. However, few HPV infections persist and progress to cervical cancer [11]. The vast majority cause no or only mild cytological abnormalities that may go undetected and regress to normalcy [11]. It is unknown why high risk HPV infection is cancerous in some women whereas in others it is eradicated. Individual differences in immunological defence may be one explanation [12]. Local cervical factors may determine the outcome of HPV. For this reason, there is a lot of interest in studying factors predisposing towards acquisition and persistence of this infection.

In contrast with cervical HPV infection, BV is associated with major changes in the vaginal environment. Because women with BV possess a *Lactobacillus*-poor flora, their changes in the vaginal ecosystem may provide biological plausibility for an increased risk or reactivation of HPV infection. Little is known about how the changed vaginal milieu in BV influences mucosal susceptibility for HPV, or vice versa, how infection with STIs in general influences the vaginal environment. The magnitude of association between BV and HPV has varied in epidemiological studies and remains controversial, yielding conflicting results and ranging from absence of any association [13] to a clear positive relationship [14].

To examine this controversial literature in more detail, a meta-analysis of available literature on the association between BV and cervical HPV infection was conducted. Estimates of association between BV and HPV are presented for HPV prevalence studies and analyzed for publication bias and heterogeneity.

## Methods

### Literature search

**Figure 1:**
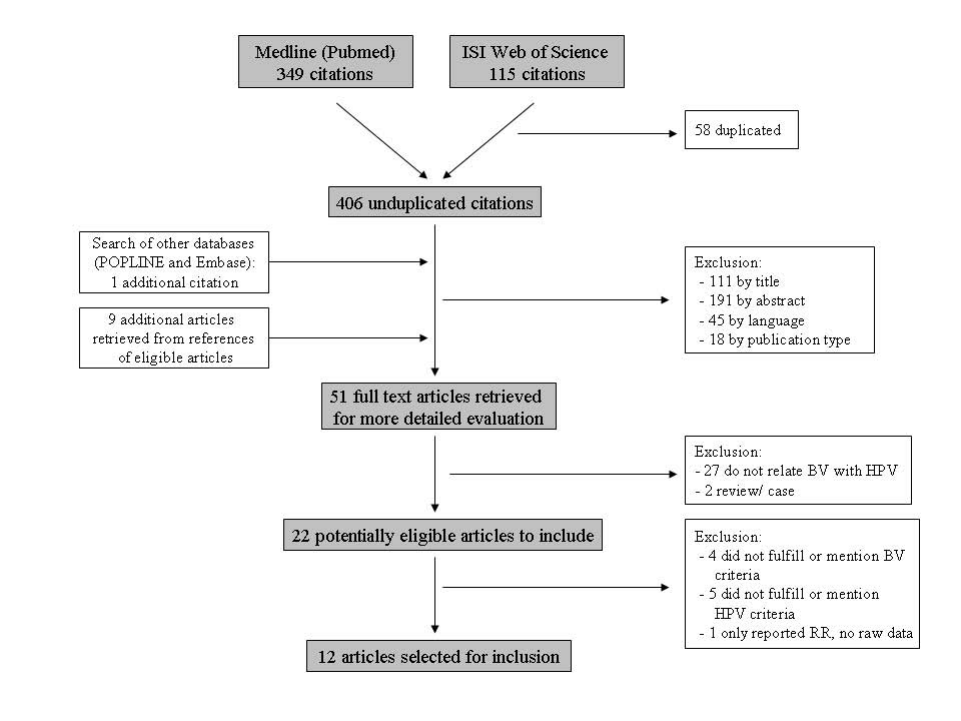
**Flow-chart of article selection for inclusion in meta-analysis BV - HPV**.

Relevant studies on association between BV and HPV infection were identified through an extensive search of Medline, based on the following keywords: 'bacterial vaginosis', 'bacterial infections or vaginitis', 'BV', 'Gardnerella', and 'dysbacteriosis', in combination with 'human papillomavirus', 'papillomavirus infections', 'HPV' or 'cervical screening'. This search yielded 349 different published articles. Web of Science was further searched using the same strings, and yielding a total of 115 different publications. Only one additional eligible article was found beyond the Medline search [15]. Studies that addressed the relationship between BV and cervical HPV infection were reviewed for predefined eligibility criteria. Two authors independently reviewed and evaluated critically all studies for inclusion (EG and DVB). Figure 1 summarizes the study selection process.

Eligible studies needed a clear description of diagnostic methods used for detecting both BV and HPV. There was no restriction in study design. Articles were included if they either reported odds ratios and corresponding 95% confidence intervals (CI) representing the magnitude of association between BV and cervical HPV infection, or presented data for calculation.

Reference lists of relevant papers and reviews were examined to identify further articles. Studies were limited to those written in English. We stopped our literature search in December 2009, but there was no publication starting-date limitation. The meta-analysis was restricted to original articles (no expert opinions, editorials or reviews). Conference abstracts and other unpublished articles were excluded, as these could not be systematically reviewed and data could not be verified. This meta-analysis was based on the Meta-analysis Of Observational Studies in Epidemiology (MOOSE) guidelines [16].

### Data abstraction and selection criteria

For each study, the following data were extracted: first author, year of publication, country where the study was conducted, study design, number of cases enrolled, study population, age range of participants, method of HPV diagnosis and HPV prevalence, BV diagnostic criteria, and BV prevalence.

Participants were categorized in four groups: women referred to colposcopy clinic because of an abnormal Pap-smear (referred), women attending family planning/obstetrics and gynaecology clinics (attendees), screening population, and mixed patient groups (referred, attendees and screened). In most studies, pregnancy was an exclusion criterion. Only one study included pregnant women [14]. Another study enrolled HIV positive and high-risk HIV uninfected women [17].

BV prevalence was recorded as an estimate of BV in the study population. Diagnostic criteria for BV included Nugent's scoring system, Amsel clinical criteria, modified Amsel criteria, and presence of clue cells. In Nugent's scoring system (BV when score ≥ 7), the most accurate method, Gram-stained vaginal smears are assessed for average number of bacterial morphotypes seen per oil immersion field (magnification 100 times). Briefly, large gram-positive rods (*Lactobacilli*) were scored inversely from 0 to 4, small Gram-variable or gram-negative rods (*Gardnerella *and *Bacteroides *spp) from 0 to 4, and curved gram-variable rods (typically *Mobiluncus *spp) scored from 0 to 2 [3]. Amsel criteria define BV as presence of any three of the following characteristics: [1] homogeneous white grey discharge that sticks to the vaginal walls; [2] vaginal fluid pH > 4.5; [3] release of fishy amine odour from vaginal fluid when mixed with 10% potassium hydroxide (positive whiff test); and [4] clue cells visible on wet mount microscopy [3]. Modification of Amsel criteria included diagnosing BV when only two of these four elements were present (Peters et al. [18] used clue cells and positive whiff test). Diagnosing BV only through presence of clue cells on wet smear or more than 20% clue cells on Papanicolaou smear was also considered an inclusion criteria, since this is confirmed by previous studies to be an accurate method and good predictor for BV [19].

Studies eligible for inclusion detected cervical HPV infection with Polymerase Chain Reaction (PCR) or Fluorescent In Situ Hybridization (FISH). Koilocytosis was not considered specific enough for HPV detection. A list of studies included in the analysis and a digest of information extracted is given in Table 1.

**Table 1:** Characteristics of the selected studies included in the meta-analysis BV - HPV

Year of publication	Authors	Country	Study Design	Nr cases enrolled	Participants	Age range (Years)	HPV Diag	HPV Prev (%)	BV Diag	BV Prev (%)
1995	Peters et al[18]	Netherlands	CS	280	referred	20 - 66	PCR	71.1	Mod Amsel	20.0
1997	Sikström et al[20]	Sweden	CS	972	attendees	-	FISH	6.8	Amsel	13.0
2001	Castle et al[21]	Costa Rica	CS	8582	screened	-	PCR	59.6	Nugent	37.8
2003	Mao et al[23]	USA	FU	516	screened	18 - 24	PCR	22.8	Amsel	3.0
2003	Boyle et al[22]	UK	CS	379	attendees	16 - 58	PCR	21.1	Amsel	30.9
2004	da Silva et al[14]	Brazil	CS	52	attendees	15 - 35	PCR	50.0	Amsel	34.6
2005	Watts et al[17]*	USA	CS	2229	attendees (HIV and high-risk)	-	PCR	56.1	Nugent	43.7
2005	Samoff et al[24]*	USA	FU	151	attendees	13 - 19	PCR	53.5	Nugent	47.2
2008	Figueiredo et al[15]	Brazil	CS	228	referred	-	PCR	84.2	Clue cells	17.0
2009	Verteramo et al[26]	Italy	CS	857	attendees	17 - 58	PCR	31.0	Amsel	6.3
2009	Nam et al[25]	South-Korea	CS	510	referred	-	PCR	69.1	Amsel	11.0
2009	Rahkola et al[13]	Finland	CS	328	mix	18 - 69	PCR	53.3	Clue cells	15.2

** Prevalence and incidence study

Abbreviations: HPV = Human Papillomavirus; BV = Bacterial vaginosis, PCR = Polymerase Chain Reaction, FISH = Fluorescent In Situ Hybridization, CS = Cross-sectional study, FU = Follow-up study, Diag = diagnosis, Prev = Prevalence

Participants: referred (women referred to colposcopy clinic because of abnormal Pap-smear), attendees (women attending family planning or obstetrics and gynaecology clinics), screened (population sample, screening), mix (referred, attendees and/or screened)

### Statistical analysis

Meta-analysis was conducted for twelve studies that fulfil the above-reported criteria [13-15,17,18,20-26], using packages for STATA provided by Sterne et al. [27]. Odds ratios and their respective standard errors were calculated from the provided raw data. For the remaining studies, crude odds ratios and standard errors as reported in the article were used [17,24]. The resulting set of odds ratios were combined into a summary estimate of the association between BV and HPV using the random effects model of DerSimonian and Laird [28] and results were visualised in a forest plot. Evidence of publication bias was ruled out by funnel plot [29] and statistically evaluated for asymmetry using the Begg rank correlation [30]. Homogeneity of effects across studies was assessed using Cochran's Q test [31] and quantified by Higgins and Thompson's I2 [32]. Relative influence of different studies was evaluated by estimating the combined odds ratio after omitting one study at a time. Cumulative analysis, in which studies were added in order of descending variance on odds ratios, was done to rule out a potential small-study effect.

## Results

### Study identification and description

Initial search gave rise to 406 unduplicated articles. Titles and abstracts were reviewed, and 51 out of 406 articles were considered of interest. These were retained for detailed evaluation, and 12 were finally retrieved for statistical analysis (Figure 1). Reasons for exclusion in the last step of our search strategy included: studies using koilocytosis as criterion for HPV detection [33-36], studies not describing their methodology of diagnosis [37,38], and studies using presence of *Gardnerella vaginalis *[39,40] or Grade II vaginal flora (according to Schröder et al.) [41] to diagnose BV.

Twelve eligible articles were identified, including a total of 6,372 women. These studies reported thirteen different estimates of association between BV and HPV prevalence for twelve study populations. One study reported estimates using two different methods of BV diagnosis, i.e. Amsel and presence of clue cells [20]. The estimate based on the most stringent method (Amsel) was used for meta-analysis.

Most studies using adjusted odds ratios (AOR) did not describe clearly potential confounders and methods used. Consequently, the reported AOR could not be compared between studies. Therefore, where possible, raw data were retrieved for statistical analysis. Two studies did not mention raw data, hence only the reported crude odds ratios could be used [17,24]. One study was excluded [42], because only crude and adjusted relative risks were described (crude RR, 1.20; 95% CI, 0.89-1.62; RR adjusted for ethnicity, sexual partners in past year and douching in past month, 1.08; 95% CI 0.82-1.42).

Studies included in the meta-analysis comprised ten cross-sectional studies [13-15,17,18,20-22,25,26] and two follow up studies [23,24]. One study measured additional incidence rates (defined as recruiting HPV-negative women and prospectively measuring incident HPV infection), but only baseline data was extracted for meta-analysis (odds ratio for incidence study, 1.41; 95% CI 1.25-1.59) [17].

Regarding geographical location, four studies were conducted in low-income [14,15,21,25] and eight in developed countries [13,17,18,20,22-24,26]). Five studies were conducted in Europe [13,18,20,26,43], three in the United States [17,23,24], three in South-America [14,15,21], and one in Asia [25]. Eligible studies performed in Africa were not found.

### Diagnosis and prevalence of bacterial vaginosis

BV was diagnosed either using clinical Amsel or modified Amsel criteria in seven out of twelve studies [14,18,20,23,25,26,43], Nugent's score in three out of twelve studies [17,21,24] and presence of clue cells in two out of twelve studies [13,15]. BV prevalence ranged from 3.0% in sexually active university students ranged 18-24 years in the USA [23] to 47.2% in sexually active women ranged 13-19 years in the USA attending a primary care clinic [24]. Large variation in reported prevalence figures can be attributed to differing recruitment strategies, inclusion of different patient populations, and variation in diagnostic criteria. Pooled BV prevalence was 31.2% (95% CI, 12.3%-51.6%).

Prevalence of BV using Nugent's criteria was consistently higher as opposed to studies using clinical Amsel criteria or presence of clue cells, ranging from 37.8 to 47.2%. Prevalence of BV using Amsel criteria and presence of clue cells ranged from 3.0 to 34.6% and from 15.2 to 20.2% respectively. Pooled prevalence of BV in low-income countries was 35.8% (95% CI, 20.8%-50.9%) while in developed countries it was 24.8% (95% CI, 12.4%-37.2%).

### Bacterial vaginosis - cervical human papillomavirus association

**Figure 2:**
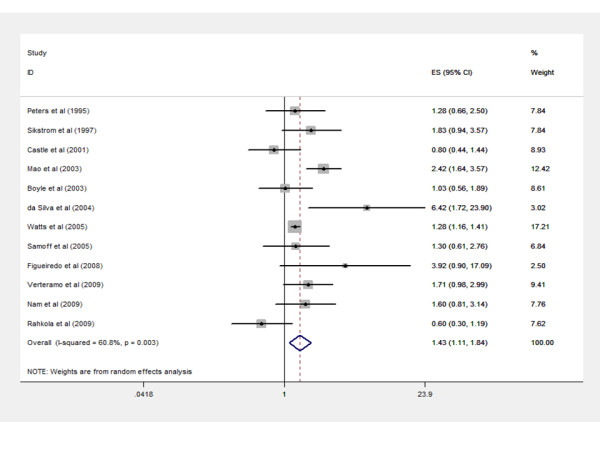
**Forest plot of estimates of association between bacterial vaginosis and cervical human papillomavirus infection**. Studies are identified by references. Each study is represented by a black square and a horizontal line, which corresponds to the estimate (ES) and 95% confidence interval (CI) of odds ratios. Area of black squares reflects weight of study in the meta-analysis.

Analysis of the association between BV and cervical HPV infection shows that HPV prevalence is significantly higher in BV positive women in only three out of twelve studies compared to women without BV [14,17,23]. Figure 2 represents reported odds ratios with their 95% CI for the likelihood of detecting cervical HPV in presence of BV, weight given to each study in random effects model, and combined odds ratio with 95% CI. Odds ratios in different studies ranged from 0.60 [13] to 6.42 [14]. The combined odds ratio for included cross-sectional studies was 1.43 (95% CI, 1.11-1.84, p = 0.005), indicating a positive association between BV and cervical HPV infection.

**Figure 3:**
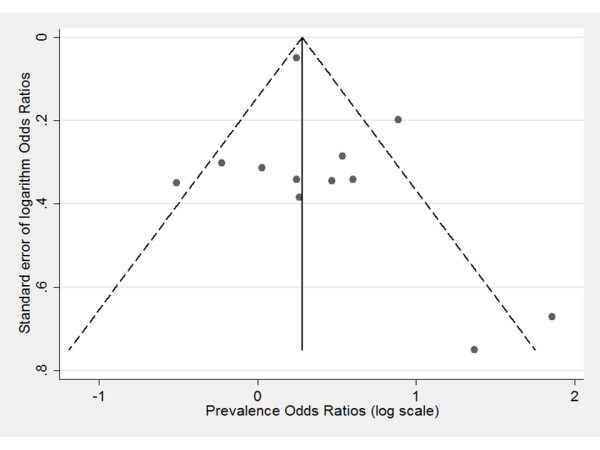
**Funnel plot to assess publication bias**. The full circles represent the 12 included study estimates of association between BV and prevalent cervical HPV infection. The size of association of each study is plotted on the horizontal axis, against the standard error on the vertical axis (on logarithmic scale). The vertical line in the funnel plot indicates the fixed-effects summary estimate, while the sloping lines indicate the expected 95% confidence intervals for a given standard error.

A funnel plot confirmed lack of obvious publication bias as no clear asymmetry could be detected (Figure 3). Also Begg's rank correlation test could not detect a significant publication bias (z = 0.82, p > 0.05). Included studies showed clear heterogeneity according to Cochran's Q test (χ^2 ^= 28.8, p < 0.01). About 60% of the total variation could be explained by heterogeneity between samples (I2 = 60.8). Two studies [14,15] reported higher odds ratios than can be expected in a homogeneous set of studies (figure 2). Substantial differences in reported odds ratios among other studies form an extra indication for existing heterogeneity.

**Figure 4:**
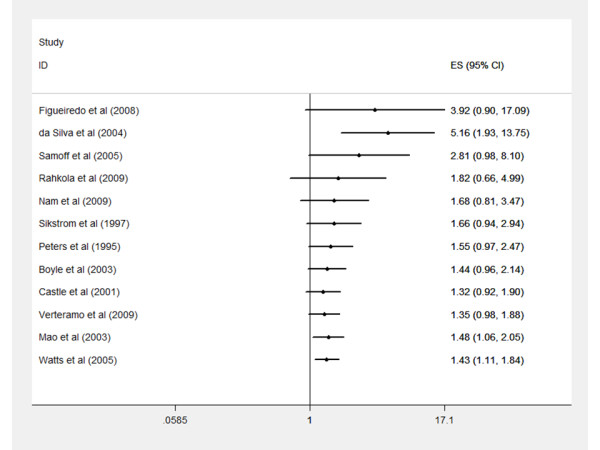
**Cumulative meta-analysis to evaluate small-study effect**. Studies are ordered according to descending variance on odds ratios. The vertical line indicates the no-association line (OR 1.0). Each study is represented by a horizontal line, corresponding to the OR (or estimates ES) and symmetric 95% CI.

Cumulative meta-analysis showed that small-study effects are unlikely to have an impact on the combined odds ratio (Figure 4). Studies with the largest standard error on their odds ratio have also a higher combined estimate [14,15]. However, this effect is only visible for two studies. In general, evolution of the combined odds ratio is stable. Moreover, two studies with an odds ratio lower than one are among the smallest in this analysis.

## Discussion

To our knowledge, this review and meta-analysis with over 6,000 women is the first systematic evaluation of association between BV and cervical HPV infection. Although BV enhances acquisition of certain STIs, its relationship to cervical HPV infection is still an issue of controversy. Our results show evidence of a positive association between these two very common conditions, with an overall estimated odds ratio of 1.43.

Several hypotheses have been postulated, supporting this association. In BV-negative women, hydrogen peroxide-producing lactobacilli dominate the vaginal microflora and are part of the main defence mechanisms [1]. Loss of these protective micro-organisms and other changes in the vaginal milieu, related to BV, could facilitate survival of other sexually transmitted agents and are risk factors for developing vaginal infections. It is well recognised that BV renders women vulnerable to acquisition of *Neisseria gonorrhoeae*, *Chlamydia trachomatis*, HSV-1 and 2, and HIV [8,9,42]. Moreover, BV has been associated with a reduction in vaginal fluid levels of secretory leukocyte protease inhibitor (SLPI), able to block HIV infection in vitro [44]. It has been documented that BV propagates viral replication and vaginal shedding of HSV, thereby further enhancing spread of this STI [45].

Another hypothesis proposes that mucin-degrading enzymes are increased in vaginal fluid of women with BV. These enzymes, like sialidases, play a role in degradation of the gel layer coating the cervical epithelium, causing micro-abrasions or alterations of epithelial cells. The team of Briselden demonstrated positivity for sialidases in 84% of BV-positive women [46]. Such enzymes may promote virulence through destroying the protective mucosa barrier and hence increase susceptibility to cervical HPV infection by facilitating adherence, invasion and eventually incorporation of HPV oncogenes into the genome of cells of the transformation zone. Abnormal vaginal microflora could also be implicated in maintenance of subclinical HPV. Furthermore, changes in cervico-vaginal milieu resulting from co-infections may exert an influence on the natural history of cervical HPV infection.

It is also possible that BV is a cofactor involved in acquisition or reactivation of HPV infection by affecting immunological balance within the cervical tissue as a result of changes in production of factors, such as cytokines (interleukin-1ß, interleukin-10) [47]. Mucosal immune system activation represents a critical response against micro-organisms colonizing the reproductive tract. Neutrophil recruitment and activation is considered the main innate immune response against microbial and viral infections of vaginal mucosa [47]. Women harbouring clue cells show no inflammatory signs and neutrophils are typically relatively absent in BV smears subjected to microscopy [15]. Enzymes produced by anaerobic bacteria involved in the pathogenesis of BV can potentially alter immune signals and promote degradation of host factors, rendering women more susceptible of acquiring HPV.

These results, however, should be interpreted in light of a number of methodological limitations. The analysis suffers from the fact that most included studies had a cross-sectional design, where data on prevalence of BV and HPV infection were gathered simultaneously, instead of over time. Therefore this analysis is liable to reverse causation bias that would result from HPV infected women being more likely to acquire BV. This disadvantage prohibits concluding that BV increases risk of HPV acquisition or that there is a causal relationship. The sequence of infection is unknown and only a follow up study can determine which condition facilitates the other. In an incidence study by Watts et al., BV was significantly associated with detection of new HPV infection at follow-up visit (OR, 1.41; 95% CI 1.25-1.59) [17]. Association between BV and HPV persisted even after adjustment for number of sexual partners, suggesting that women with BV may be more susceptible for HPV and not simply because of shared risk factors. In contrast, another longitudinal study performed a time-lag analysis to evaluate which condition preceded the other [23]. The result suggested a temporal relationship, where BV was found to occur simultaneously with or after HPV infection, rather than ante-dating acquisition of HPV. Perhaps cervical HPV infection may favour changes in the vaginal milieu that enhances development of BV.

The question remains whether BV and cervical HPV infection are simply related because there is a biologic interaction between them, or because both occur frequently in sexually active women. A positive correlation between BV and HPV might be explained by the fact that sexual risk behaviour and promiscuity are found more often in women with BV than in comparison groups. Role of sexual transmission in causing or promoting BV continues to be a topic of debate, as e.g. highlighted by data in lesbians, who have a high prevalence of BV [48]. Although not considered an STI in its usual sense (e.g. treatment of the sexual partner has no effect on frequency or relapses), the epidemiological profile of BV mirrors an STI [49]. HPV is known to be one of the most common STIs, thus concerns regarding confounding by sexual behaviour certainly remain.

A number of variables are contributing to observed heterogeneity. Most prominent, prevalence of BV varied according to the population studied. Various social habits and ethno-geographical risk factors may explain the wide BV prevalence range observed (3%-47.2%). It is well recognized that prevalence of BV in African women is among the highest worldwide [1]. This meta-analysis did not include studies conducted in Africa. Considering the high prevalence of BV in this continent, it would be very interesting to evaluate the association between BV and cervical HPV infection in African women, since we may expect a more pronounced effect. Our unpublished data of a cross-sectional study including 820 HIV-negative female sex workers in Mombasa (Kenya) confirms this. In multivariate logistic regression, controlled for other STIs and behavioural characteristics, borderline significance was found between BV and high-risk HPV infection (AOR, 1.72; p = 0.06).

Technical biases (e.g. collection of specimen), subjectivity, sensitivity and specificity of diagnostic methods are also attributing to detected heterogeneity. HPV detection methods varied among included studies (e.g. FISH is less sensitive compared to PCR) and also distribution of HPV viral genotypes differed largely. However, high-risk genotypes 16 and 18 present in prophylactic vaccines were (when mentioned) always included.

Further, this meta-analysis was limited to that of published studies, which could have caused publication bias, resulting from tendency to selectively publish results that are statistically significant. However, this had probably little impact as there was no evidence of funnel plot asymmetry. In addition, most studies reported a non-significant effect, which makes publication bias highly unlikely.

Currently available vaccines targeting HPV types 16 and 18, accounting for 70% of cervical cancers worldwide, opened up new avenues in prevention of this important public health problem. If a longitudinal prospective study shows a cause - effect model, than it is clear that greater attention needs to be given to BV in the global fight against HPV infection and women with BV should be considered a priority group for prophylactic vaccination. Cervical screening remains of course a major preventive focus for the cancer control program. If BV is a risk factor for cervical HPV acquisition, it is clear that screening guidelines must adapt and implement a sensitive tool like HPV DNA testing in primary screening in BV-positive women, instead of cytological testing. Closer follow-up of these patients should be considered. Restoring the vaginal microflora should in that case be a promising answer to the high prevalence of HPV infections. Randomized clinical trials to determine effect of BV control measures on HPV acquisition may then be worth considering. In addition to the need to evaluate the potential of BV treatment to prevent HPV acquisition and transmission, a better understanding of its risk factors and determinants of recurrence is required.

## Conclusion

This meta-analysis suggests a positive association between BV and cervical HPV infection. Considering that these conditions are very common among women worldwide, further research in this field is imperative. More data from prospective studies are needed to accurately evaluate temporal sequence of acquisition of both conditions in any attempt to determine a causal relationship and to identify specific sub-populations with a stronger association between BV and HPV.

## Competing interests

The authors declare that they have no competing interests.

## Authors' contributions

DVB and MT created the concept and design of this study. EG and DVB were responsible for literature search and extraction of data. JM carried out the statistical analysis. EG and DVB drafted the manuscript, which was critically revised and edited by HV, CB, PDS and MT. All authors read and approved the final manuscript.

## Pre-publication history

The pre-publication history for this paper can be accessed here:

http://www.biomedcentral.com/1471-2334/11/10/prepub
